# Role of Transthoracic Lung Ultrasonography in the Diagnosis of Pulmonary Embolism: A Systematic Review and Meta-Analysis

**DOI:** 10.1371/journal.pone.0129909

**Published:** 2015-06-15

**Authors:** Libing Jiang, Yuefeng Ma, Changwei Zhao, Weifeng Shen, Xia Feng, Yongan Xu, Mao Zhang

**Affiliations:** 1 Department of Emergency Medicine, Second Affiliated Hospital, School of Medicine &Institute of emergency Medicine, Zhejiang University, Hangzhou, China; 2 China Emergency and Critical Care Evidence-based Group, Hangzhou, China; 3 Department of respiration, The Third People’s Hospital of Hangzhou,Hangzhou, China; Kurume University School of Medicine, JAPAN

## Abstract

**Background:**

Pulmonary embolism (PE) is a potentially life-threatening condition. Although computed tomography pulmonary angiography (CTPA) is the reference standard for diagnosis, its early diagnosis remains a challenge, and the concerns about the radiation exposures further limit the general use of CTPA. The primary aim of this meta-analysis was to evaluate the overall diagnostic accuracy of transthoracic lung ultrasound (TLS) in the diagnosis of PE.

**Methods:**

PubMed, Web of science, OvidSP, ProQuest, EBSCO, Cochrane Library and Clinicaltrial.gov were searched systematically. The quality of included studies was assessed using the Quality Assessment of Diagnostic Accuracy Studies-2 tool. The sensitivity, specificity, positive likelihood ratio (PLR), negative likelihood ratio (NLR), diagnostic odds ratio (DOR) and hierarchical summary receiver operating characteristic (HSROC) curves were used to examine the TS performance. The Bayes analysis was used to calculate the post-test probability of PE. Publication bias was assessed with Deeks funnel plot.

**Results:**

The results indicated that the sensitivity, specificity, PLR and NLR were 0.85 (95% confidence interval (CI), 0.78 to 0.90), and 0.83 (95% CI, 0.73 to 0.90). And the DOR and HSROC were 28.82 (95% CI, 17.60 to 47.21), 0.91(95% CI, 0.88, 0.93).

**Conclusions:**

The present meta-analysis suggested that transthoracic lung ultrasonography is helpful in diagnosing pulmonary embolism. Although the application of transthoracic lung ultrasound may change some patients’ diagnostic processes, it is inappropriate to generally use transthoracic ultrasonography in diagnosing pulmonary embolism currently.

## Introduction

Pulmonary embolism (PE) is a life-threatening clinical emergency [1, 2]. It is estimated that at least 600,000 cases of PE are diagnosed per year in the United States [1]. When treated, PE has a mortality rate of 2%-8%, but when left untreated, the mortality rate is as high as 25%-30% [2, 3].Up to now, the mortality from PE remains high because of its difficult diagnosis [1–8].

Pulmonary angiography (PA) has long been taken as the gold standard for diagnosis of PE,however invasive, costly and sometimes difficult it is to get a clear conclusion [4]. Therefore, some non-invasive methods have been used for early diagnosis of PE, especially computed tomography pulmonary angiography (CTPA). In recent years, CTPA has replaced PA as a first-line method for the diagnosis of PE, because of its easy availability, high accuracy and ability of differential diagnosis [6, 9]. However, only 13%-33% of patients with suspected acute PE are tested positive for acute PE on CTPA [10, 11]. Meanwhile, the use of CTPA results in significant exposure to ionizing radiation, as well as a risk of contrast nephropathy. In addition, in most hospitals, particularly in developing countries, CTPA cannot be performed directly in the emergency room [11].

In recent years, a growing number of studies have demonstrated that ultrasound can be used for the diagnoses of various pulmonary, pleural, and mediastinal pathologies [2, 6]. However, the diagnostic value of ultrasound of PE is unclear [12–19]. The objective of this meta-analysis was to evaluate the overall accuracy of the transthoracic lung ultrasound (TLS) in the diagnosis of PE.

## Materials and Methods

### Study design and data sources

We searched PubMed, Web of science, OvidSP (EMBASE), ProQuest, EBSCO, Cochrane Library and Clinicaltrial.gov for relevant studies up to Oct 2014. The following free text words or medical subject headings were used alone or in combination: ultrasound, sonography, ultrasonography, pulmonary embolism, PE, pulmonary thromboembolism, PTE, pulmonary infarction, VTE, venous thromboembolism, sensitivity, and specificity. In addition, the reference lists of all eligible articles and reviews were also screened for additional studies. There was no language restriction.

### Study selection

#### Inclusion criteria

Eligible studies were selected and examined independently by two investigators (LBJ and YFM) according to the following inclusion criteria:

1. Population: Adult patients with suspected acute PE (not a case-control design).
2. Intervention: TLS, CTPA, PA, MRI etc. were used for the diagnosis of PE independently.
3. Comparison: the diagnostic accuracy of TLS was evaluated compared to the gold standard of any type for diagnosing PE (CTPA, ventilation/perfusion (V/P) scintigraphy or a combination of several diagnostic approaches, and noteworthy, TLS should not be a component of the gold standard).
4. Outcomes: true positives, false positives, false negatives and true negatives. If not directly reported, these data had to be retrievable through calculation.
5. Type of study: diagnostic test.

### Assessment of methodological quality

The Quality Assessment of Studies of Diagnostic Accuracy included in Systematic Reviews-2 (QUADAS-2) tool (http://www.bris.ac.uk/quadas/quadas-2/), which is recommended by the Cochrane Diagnostic Test Accuracy Working Group, was used to assess the methodological quality of all included studies. This tool is composed of two parts: the risks of bias and concerns regarding applicability. The former was assessed in four domains: patient selection, index test, reference standard, and flow and timing. And the latter was assessed in three domains: patient selection, index test, and reference standard. This process was performed using the Review Manager 5 (http://ims.cochrane.org/revman/download).

### Data extraction

The following data items were extracted by two investigators (XF and LBJ) independently using a specific data extraction sheet: characteristics of studies, characteristics of patients, the ultrasonic diagnosis criteria for PE, reference standards, study quality and outcomes. Discrepancies were resolved by consensus.

### Data analysis

Sensitivity, specificity, likelihood ratios (LR) and diagnostic odds ratio (DOR) were pooled using the bivariate mixed-effects binary regression model. The area under the summary receiver operating characteristic (sROC) curve (AUC) was calculated [20]. We also constructed hierarchical summary receiver operation characteristic (HSROC) curves to summarize the global text performance from different diagnostic studies [21]. The 95% confidence region and the 95% prediction region around the pooled estimates were plotted to illustrate the precision with which the pooled values were estimated (confidence ellipse around the mean value) and to illustrate the amount of between-study variation (prediction ellipse) [21]. The between-study heterogeneity was evaluated with the I^2^ statistic and Chi-square test using the State software. *p*<0.1 or *I* ^*2*^>50% suggested notable heterogeneity [22]. In test accuracy studies, threshold effect is one of the primary causes of heterogeneity. The Spearman correlation coefficient between the logit of sensitivity and the logit of (1-specificity) was calculated to assess the threshold effect using Meta-Disc version 1.4 (http://www.hrc.es/investigacion/metadisc_en.htm). A strong positive correlation (P<0.05) would suggest a threshold effect [22]. Publication bias was inspected by a Deeks funnel plot of the diagnostic odds ratio against study size [23].

We used Bayes theorem to estimate the post-test probability of PE, by multiplying the pretest odds by the likelihood ratio, where pretest odds are calculated by dividing the pre-test probability by (1-pre-test probability). Then the post-test probability equals post-test odds divided by (1+ post-test odds) [24]. We evaluated the pretest probabilities of 1.3%, 16.2%, and 40.6% versus corresponding post-test probabilities following a “positive” or “negative” TS result, based on the summary sensitivity and specificity using Fagan plot analysis [24]. The magnitudes of low (1.3%), moderate (16.2%) and high pre-test probability (40.6%) were defined according to the Wells’ Criteria (http://www.mdcalc.com/wells-criteria-for-pulmonary-embolism-pe/).

Studies included in our meta-analysis were published as long ago as 1990. As we all know, ultrasound technology and understanding have changed immensely since then; thus, the following subgroup analysis was created: comparisons between studies published pre-2000 and post-2000.

All statistical analyses were performed using the MIDAS and METANDI modules in STATA software, version 12.0 (SERIAL NO.40120519635).

## Results

### Study identification and characteristics

**Fig 1:**
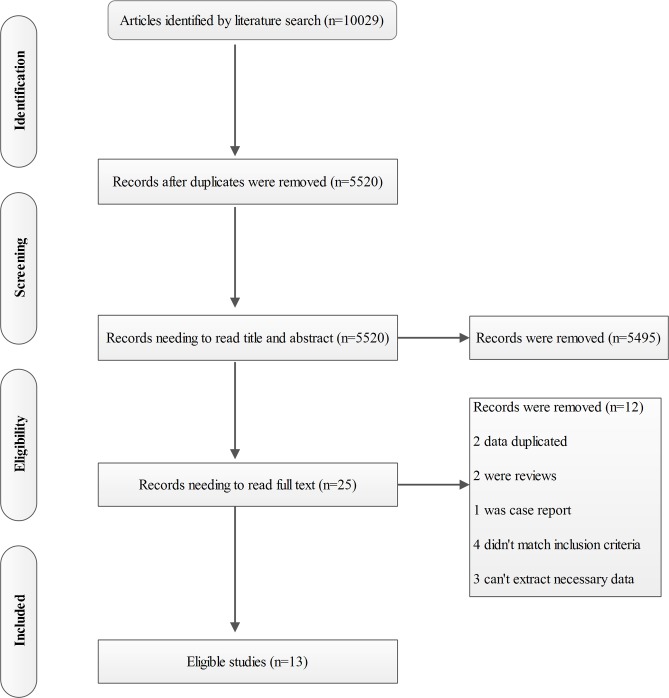
Flow chart of study selection for inclusion in the systematic review.

Thirteen studies were eligible for this meta-analysis [10, 17–19, 25–33]. The flow chart and according exclusion reasons were summarized in **Fig 1**. The sample sizes ranged from 33 to 357, and a total of 1356 patients (mean age, range 54.1–71 years; male sex, range 25–61%) were included. The detailed characteristics of included studies are showed in **Table 1**and **S1 Table.**

**Table 1:** Characteristics of Eligible Studies-1.

Author	Year	Patients (*n*)	Male (%)	Consecutive	Age	TP	FP	FN	TN	Ultrasonography technology
Peiman [10]	2013	357	47	1^†^	71 (14) ^§^	67	10	43	237	4–8 MHz liner probe /3.5–5 MHz curved probe
Comert [24]	2013	50	54	1^†^	54.1 (17.9) ^§^	27	8	3	12	3.5 Mhz convex probe
Pfeil[28]	2010	33	52	2^‡^	65.4 (19–92)^¶^	7	7	3	16	5/3.5 MHz convex probe
Mathis[23]	2005	352	47	2^‡^	64 (18–98)^¶^	144	8	50	150	3.5–6 MHz curved array /sector scanner
Reissing[31]	2004	62	60	1^†^	62.2 (24–88)^¶^	30	2	9	21	5 / 3.5 MHz convex scanner
Mohn[25]	2003	74	57	1^†^	66 (17) ^§^	22	10	9	33	5-MHz linear probe
Lechleitner[26]	2002	55	25	1^†^	69 (23–91)^¶^	29	1	7	18	3.75 MHz sector scanner, 7.5 MHz/10.0MHz linear scanner
Reissing[27]	2001	69	61	1^†^	62.8 (24–88)^¶^	35	2	9	23	5 MHz/ 3.5 Mhz convex scanner
Mathis[19]	1999	117	58	2^‡^	NR	66	6	4	41	3.5 MHz convex probe
Lechleitner[18]	1998	67	NR	1^†^	66 ^§^	18	15	3	31	3.75 MHz sector scanner and a 7.5 MHz linear scanne
Mathis[17]	1993	54	54	1^†^	63 (21–88)^¶^	41	4	1	8	5 MHz sector scanner
Kroschel[30]	1991	33	NR	2^‡^	59 (17–85)^¶^	28	1	3	1	3.5/ 5 MHz curved / liner probe
Mathis[29]	1990	33	NR	1^†^	NR	27	2	1	3	5 MHz sector scanner

NR, not reported

§, mean(SD)

¶, median(range)

TP = true positive; FP = false positive; FN = false negative; TN = true negative

1^†^, = yes

2^‡^, = no.

### Assessment of methodological quality

**Fig 2:**
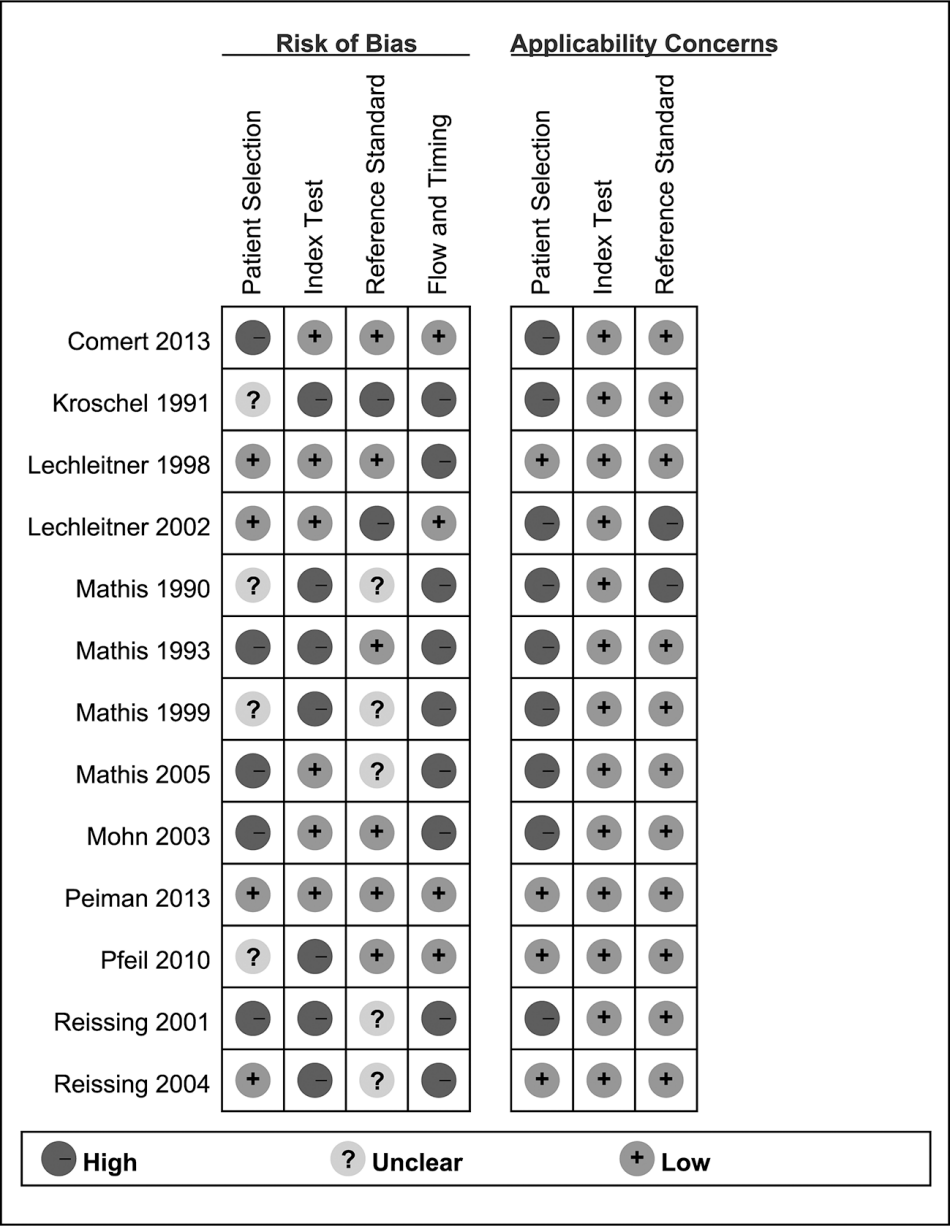
Summary of QUADAS-2 assessments of included studies.

**Fig 2**showed the quality assessment of individual studies.

### Meta-analysis

**Fig 3:**
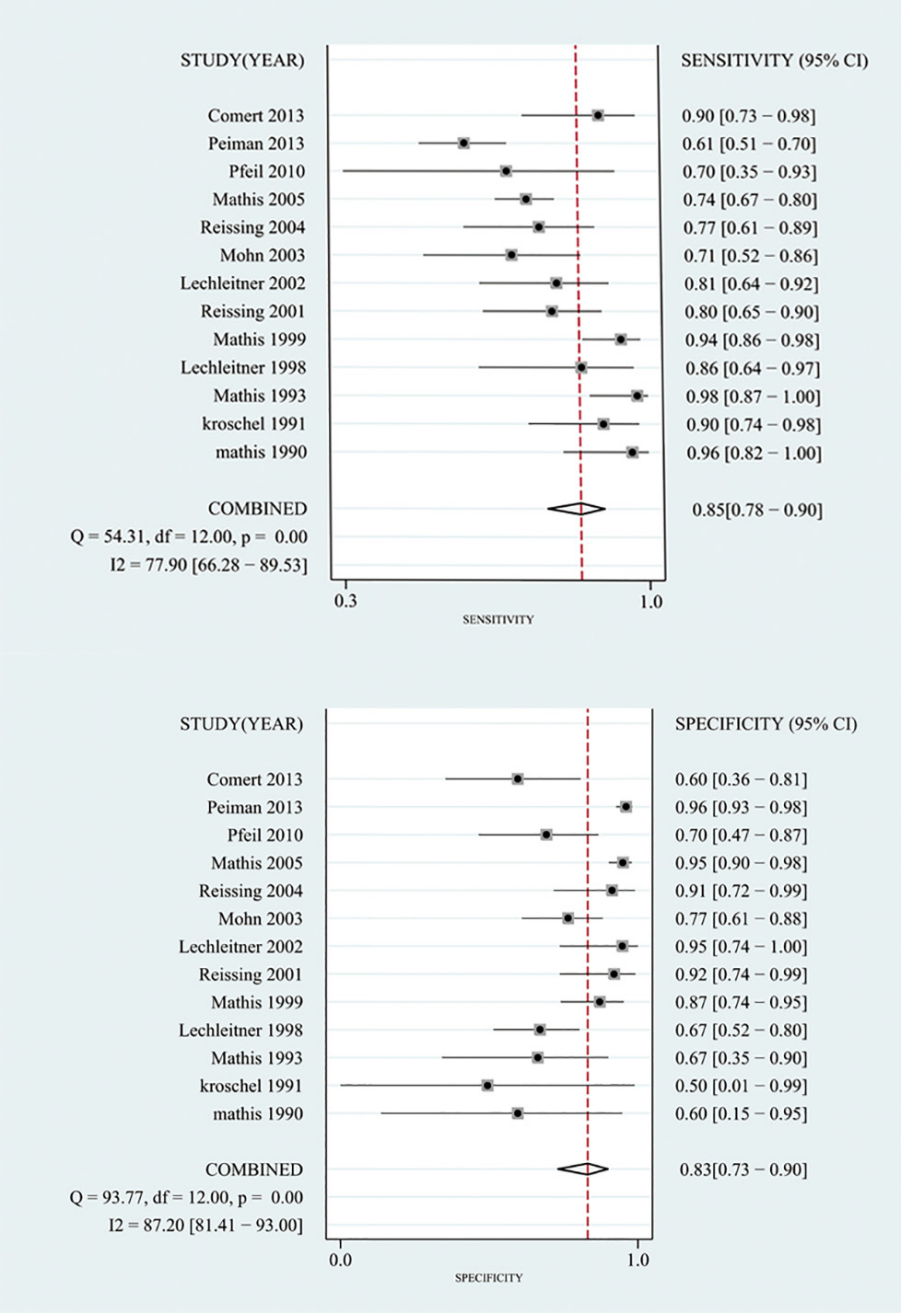
Forest plot for sensitivity and specificity.

**Fig 4:**
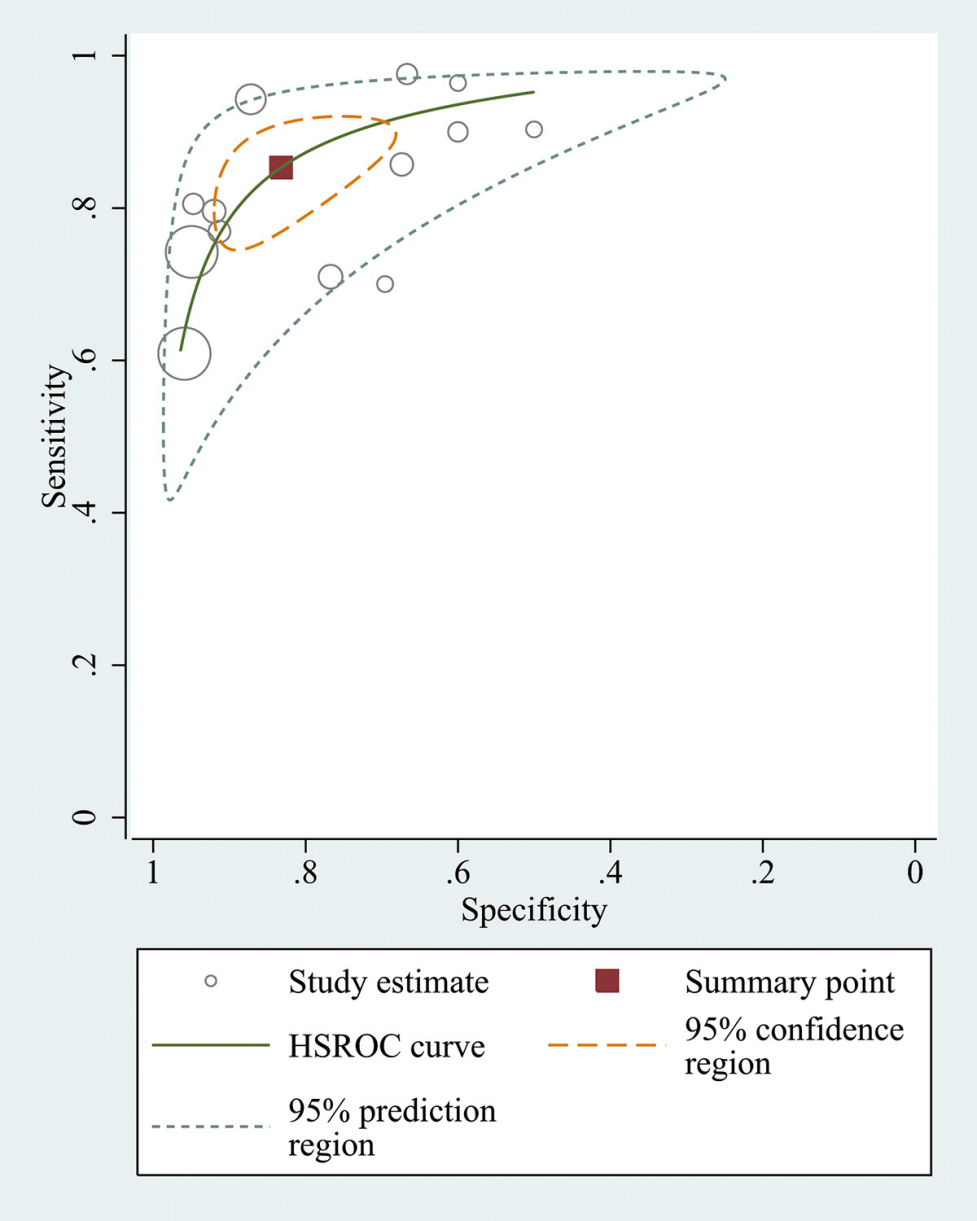
Hierarchical summary receiver operating characteristic (HSROC) curves for the detection of pulmonary embolism using transthoracic ultrasonography.

**Fig 5:**
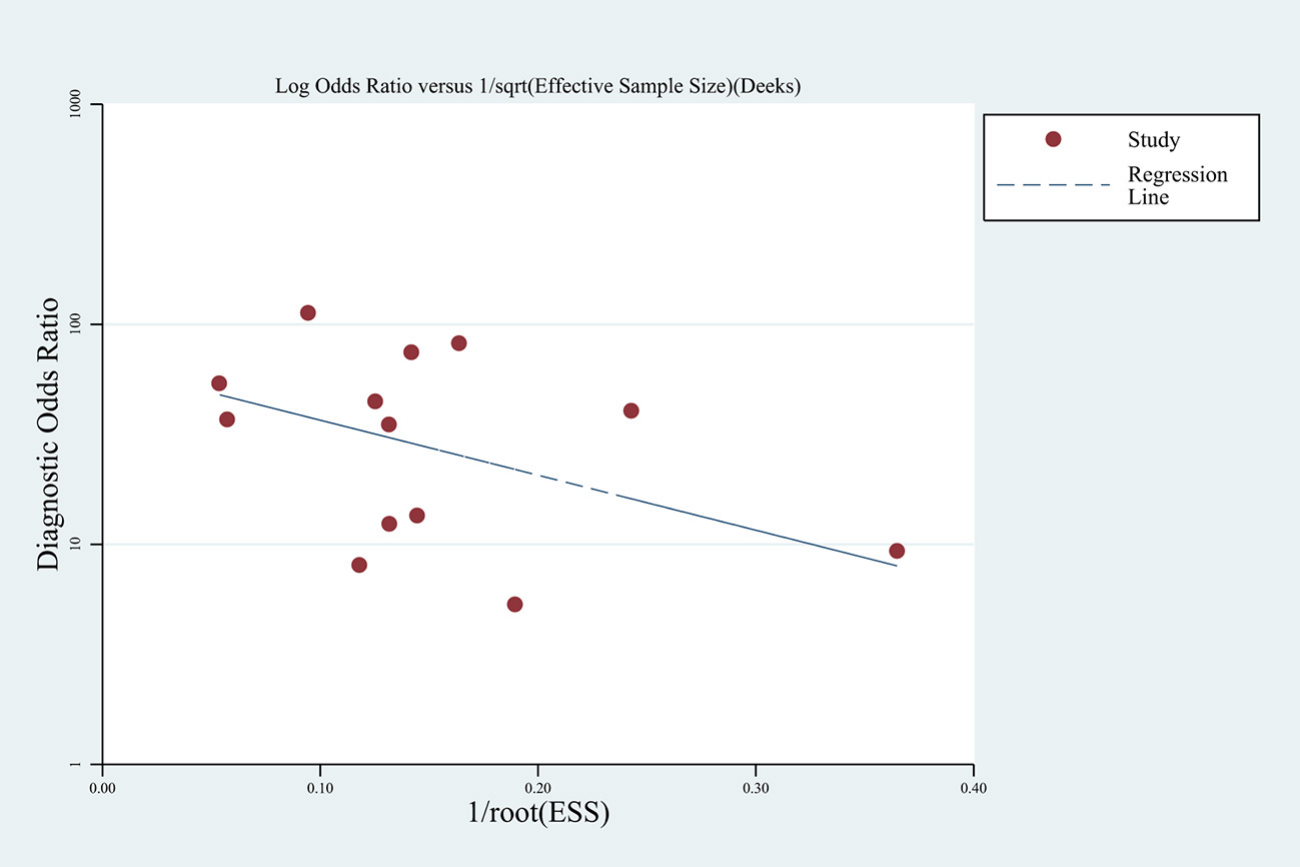
The Deek’s funnel plot for the assessment of potential publication bias.

The summary sensitivity and specificity values were 0.85 (95% confidence interval (CI), 0.78 to 0.90), and 0.83 (95% CI, 0.73 to 0.90) (**Fig 3**). The summary PLR, NLR and DOR were 5.09 (95% CI, 3.25 to 7.97), 0.18 (95% CI, 0.12 to 0.25) and 28.82 (95% CI, 17.60 to 47.21). The HSROC was 0.91 (95% CI, 0.88 to 0.93) (**Fig 4**). The Deeks funnel plot asymmetry test showed there was no evidence of significant publication bias (*p* = 0.197) (**Fig 5**).

There was significant heterogeneity among included studies, and therefore, random-effect model was used to pool these results. A Spearman rank correlation was determined to be 0.652 (*p* = 0.02), which indicated that there was significant threshold effect among individual studies. Given the various ultrasonic criteria for the diagnosis of PE among our included studies, the significance of threshold effect was not surprising.

### Bayes analysis

**Fig 6:**
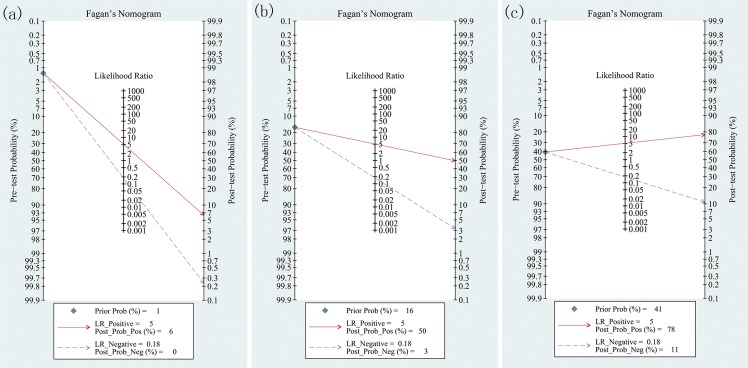
Fagan plot analysis to evaluate the clinical utility of transthoracic ultrasonography (TS) for the detection of pulmonary embolism (PE). (a) With a pretest probability of PE of 1.3%, the post-test probability of PE, given positive and negative TS results, were 6% and 0%. (b) With a pretest probability of PE of 16.2%, the post-test probabilities of PE, given positive and negative TS results, were 50% and 3%. (c) With a pretest probability of PE of 40.6%, the post-test probability of PE, given positive and negative TS results, were 78% and 11%. The Fagan plot consists of a vertical axis on the left with the pretest probability, an axis in the middle representing the likelihood ratio, and a vertical axis on the right representing the post-test probability.

We evaluated the pretest probabilities of 1.3%, 16.2%, and 40.6% which were defined according to the Wells’ Criteria versus corresponding post-test probabilities following a “positive” or “negative” TLS result, based on the summary sensitivity and specificity using Fagan plot analysis [34, 35]. This allowed the determination of the relationship between the prior defined probability, the likelihood ratio, and posterior test probability. The Fagan plot analysis suggested that the TLS had only 6% and 50% probabilities of correctly detecting PE following a “positive” TLS result when pretest probabilities were 1.3% and 16.2%, respectively (**Fig 6**). And TLS was informative lowering the negative post-probability of PE to as low as 0% and 3% when “negative” measurement from 1.3% and 16.2% pre-probability; Although TLS was very informative, with a 78% probability of correctly detecting PE following a “positive” TLS result when pretest probability was 40.6%, the diagnosis would be wrong in 11% of patients with a “negative” measurement (**Fig 6**).

**Fig 7:**
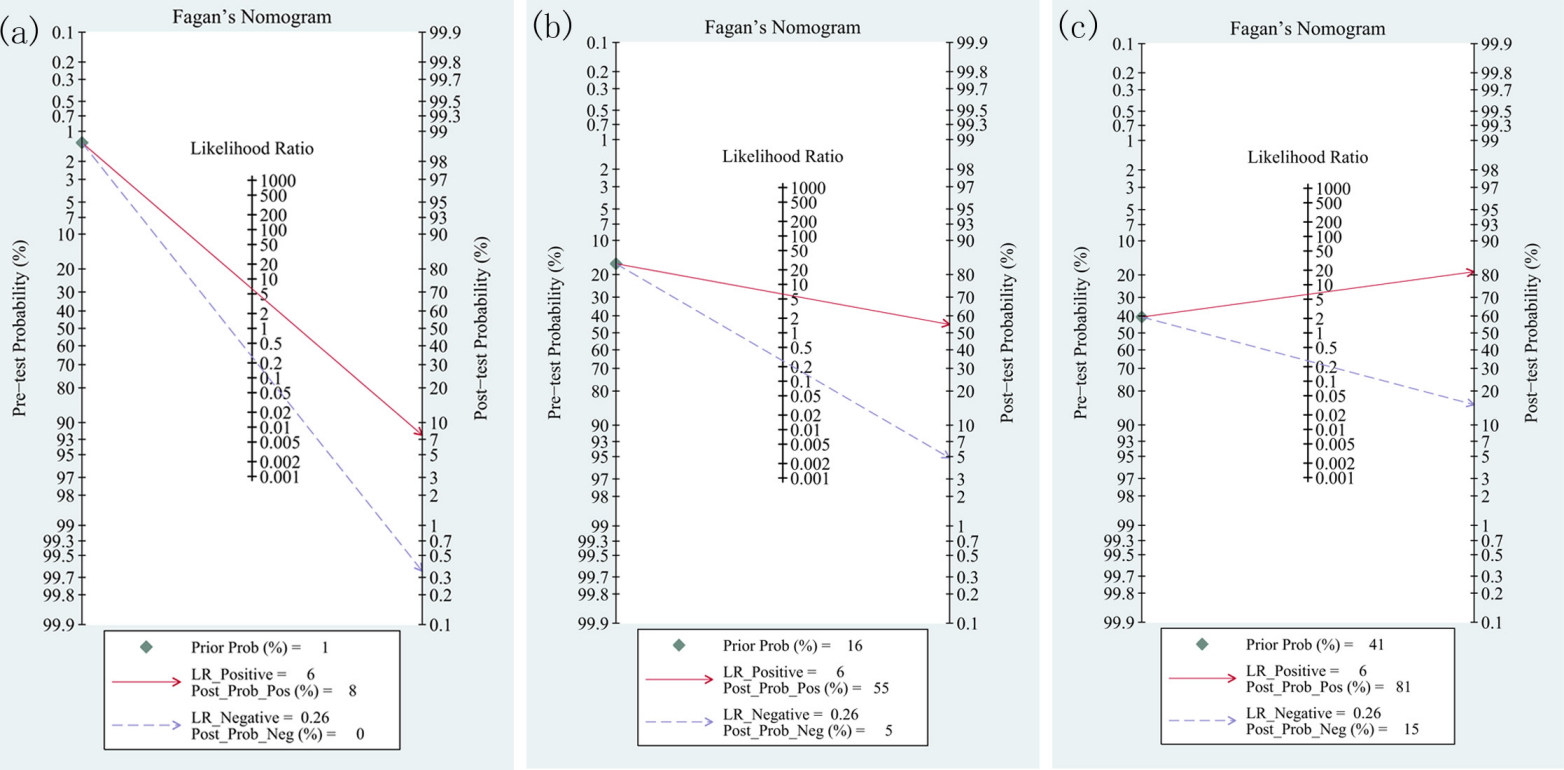
Fagan plot analysis to evaluate the clinical utility of transthoracic ultrasonography (TS) for the detection of pulmonary embolism (PE) from studies published post-2000. (a) With a pretest probability of PE of 1.3%, the post-test probability of PE, given positive and negative TS results, were 8% and 0%. (b) With a pretest probability of PE of 16.2%, the post-test probabilities of PE, given positive and negative TS results, were 55% and 5%. (c) With a pretest probability of PE of 40.6%, the post-test probability of PE, given positive and negative TS results, were 81% and 15%. The Fagan plot consists of a vertical axis on the left with the pretest probability, an axis in the middle representing the likelihood ratio, and a vertical axis on the right representing the post-test probability.

**Fig 7**showed the relationship between pretest and post-test probability based on the likelihood of a positive (above diagonal line) or negative (below diagonal line) test result over the 0–1 range of pretest probabilities. For a test with high predictive value the curve for positive results would be close to the top of the graph and the curve for negative results close to the bottom. The **Fig 7**shows that across most underlying prevalence values the TLS result does not discriminate well and generally leaves uncertainty about the presence of PE, that is additional methods which would be required to diagnose or exclude PE. In the few scenarios (pre-test probability values above 60% or below 12%) where the post-test probability of PE after a positive TLS result was large enough to diagnose PE accurately or the post-test probability after a negative result was sufficiently low to rule out the diagnosis. This conclusion was based on the hypothesis that a strategy was accurate enough to diagnose PE when the post-test probability was above 85%, and a strategy was safe enough to exclude PE when the post-test probability was below 3% [35]. Meanwhile, a positive TLS result increased the probability of PE by average 34.4%, and a negative test result reduced this by average 30% among moderate risk populations.

### Subgroup analysis

**Fig 8:**
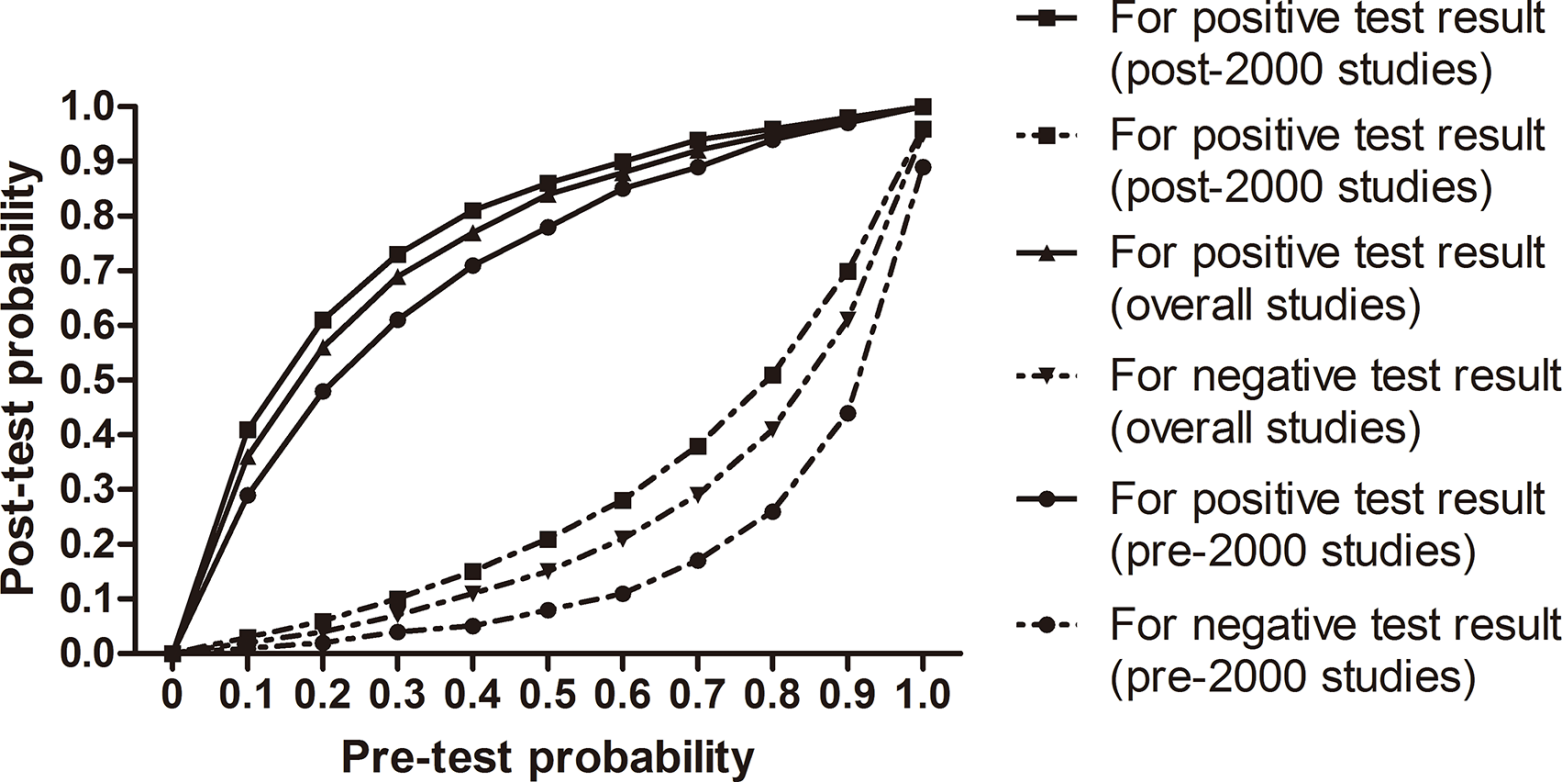
The relationship between post-test probability and pre-test probability.

There were 5 studies published pre-2000 [17–19, 31, 32], and the pooled sensitivity, specificity, PLR, NLR and DOR were 0.94 (95% CI, 0.89 to 0.96), 0.74 (95% CI, 0.61 to 0.85), 3.64 (95% CI, 2.26 to 5.84), 0.09 (95% CI, 0.05 to 0.16) and 42.31 (95% CI, 17.15 to 104.4). The HSROC was 0.95 (95% CI, 0.92 to 0.96). There were 8 studies published post-2000 [10, 25–30, 33], and the pooled sensitivity, specificity, PLR, NLR and DOR were 0.77 (95% CI, 0.70 to 0.83), 0.88 (95% CI, 0.78 to 0.94), 6.31 (95% CI, 3.56 to 11.19), 0.26 (95% CI, 0.21 to 0.34) and 23.89 (95% CI, 13.44 to 42.46). The HSROC was 0.86 (95% CI, 0.83 to 0.89). We evaluated the pretest probabilities of 1.3%, 16.2%, and 40.6% versus corresponding post-test probabilities following a “positive” or “negative” TLS, based on the summary likelihood ratio which was calculated from studies published post-2000 (**Fig 7**). When pretest probability was 1.3% a positive test result would increase the probability of PE to 8% and a negative test result would reduce the probability to 0%; When pretest probability was 16.2% a positive test result would increase the probability of PE to 55% and a negative test result would reduce the probability to 5%; When pretest probability was 40.6% a positive test result would increase the probability of PE to 81% and a negative test result would reduce the probability to 15%. The relationship between pre-test and post-test probability based on the likelihood of a positive (above diagonal line) or negative (below diagonal line) test result over the 0–1 range of pretest probabilities was summarized in **Fig 8.**

## Discussion

The present meta-analysis reveals that the TLS is helpful in the diagnosis of PE. The AUC was 0.91 and the pooled sensitivity, specificity and DOR were 85%, 83% and 28.82 respectively. A positive or negative TLS result could increase or reduce the probability of PE by nearly 30% among moderate risk populations, whereby some patients’ diagnostic processes may be changed. Although our results suggested that in few scenarios the positive or negative TLS result would diagnose or exclude PE accurately, up to date, there are no tools for calculating the appropriate pre-test probability.

PE is a potential life-threatening emergency which requires timely diagnosis and adequate anticoagulant therapy [1, 2, 6]. When left untreated, the mortality rate is as high as 30% [2, 3, 5]. However, the early diagnosis of PE remains a big challenge due to its diverse, non-specific and sometimes silent clinical features [1, 2, 6]. Therefore, many different methods for detecting PE have been proposed such as PA, V/P scintigraphy, echocardiography, lower-limb compression ultrasound (CUS), Magnetic resonance angiography (MRA) and D-dimer test [2, 4, 5, 34, 35, 36]. However, these methods were limited in their invasive characteristic, or its low sensitivity or specificity [2, 4, 5, 35, 36]. In recent years, CTPA has replaced PA as the gold standard for the diagnosis of PE [2]. It has been reported that the use of CT scan has increased from 3 to 70 million between 1980 and 2007 [11]. Whereas in patients undergoing CTPA for suspected PE, the prevalence of PE is only 5% to 10% in America and 20% to 30% in Europe [37–39]. CTPA is costly and cannot be fully available 24 hours a day, 7 days a week in the emergency department, especially in the developing countries. In addition, it is associated with an increased risk of radiation exposure [2, 4, 5]. In the United States, 1.5%-2.0% of malignant tumors are associated with CT scan and the incidence of contrast-induced nephropathy after having CTPA in patients suspected of PE is up to 12% [11]. Therefore, we have a responsibility to find new diagnostic tools to reduce the number of unnecessary CTPA, such as TLS.

The air-containing lung parenchyma has long been considered as a poor sonic window [2]. However, the development of lung consolidation and pleural effusion significantly extend the application scope of TLS [2, 24]. TLS has several advantages in the diagnosis of PE. Firstly, the pathological changes of PE are a dynamic process. It has been reported that an embolus can decrease in size within 48 h and even completely disappear after 2 days due to local fibrinolysis [40]. Based on the above analysis, along with its unspecific symptoms, some pulmonary thrombus may not be detected by angiography or CTPA due to the lag period. However, this dynamic process can be captured by TLS because it can be applied early and repeatedly [2, 4–6].Secondly, the resolution of ultrasonography in the sub-pleural region of pulmonary parenchyma is better than CTPA. Thus, smaller pulmonary embolus, which is located in the peripheral lung parenchyma near the pleural surface, can be detected by TLS [26]. Thirdly, sonography is acknowledged to be the most sensitive tool for the detection of pleural effusions which is a common feature of PE and is detectable in up to 57% of the cases on CT [41]. This will increase the diagnostic accuracy of TLS. Finally, TLS is a non-invasive method, so it doesn’t involve unnecessary radiation expose [42].

However, TLS is not without limitation: 1) TLS can detect embolic parenchymal lesions only after these lesions extend up to the peripheral lung. Although this is often the case, there is a small number of cases in which only the centrally located vessels are affected [6]. As for central PE (the thrombotic occlusion of the pulmonary trunk or of a main or a lobar pulmonary artery [43]), endobronchial ultrasound (EBUS) may be useful. The pulmonary vessels parallel the central airways at a distance of less than 5 mm, and there are no ventilated tissues between them and the bronchi makes EBUS a feasible technology to detect central PE [44]. Additionally, fatal emboli are always central PEs, and 90% of patients with fatal emboli die in the first 2 h following the occurrence of the first symptoms, therefore the diagnostic procedure must be fast and accurate [45,46]. In a single patient by Casoni and in a case series of 8 patients by Senturk, the authors all reported that EBUS can be used for the diagnostic imaging of central cases of PE [47, 48]. Subsequently, Aumiller and colleagues undertook a pilot study to evaluate the presence of PE using EBUS in 32 CT-confirmed central PE patients at the bedside within 24 hours of diagnosis. And the authors detected 97/101 emboli seen on CT which were sufficient to diagnose all these 32 patients with central PE [46]. Based on the above analysis, EBUS may be an emerging new tool to diagnose PE, especially in patients with renal failure or patients allergic to contrast or patients who are pregnant or hemodynamically unstable patients. And in the next step, EBUS may be used to measure the pulmonary hypertension, which is a complication of PE [49]. Moreover, concerns about radiation and contrast media, inherent in computed tomography pulmonary angiography could be avoided in EBUS process; therefore it is suitable for repeated short-term follow up. 2) Nearly one third of peripheral lung areas are covered with bony structures and are not accessible to TLS. However, most emboli occur in the lower lobes where are readily accessible to TLS [6]; 3) TLS is a subjective and operator-dependent tool [6, 9].

Our study had several strengths when compared to the study by Squizzato et al [9]. Firstly, more studies were included in the present study; Secondly, in the study by Squizzato et al, the authors only reported mean sensitivity and specificity. However, in the present meta-analysis, we reported more clinically relevant indicators including likelihood ratio, DOR, AUC and post-test probability of PE. Thirdly, as we all know, ultrasound technology and understanding have changed immensely since 1990, and thus, a pre-defined subgroup analysis was created in our study: comparisons between studies published pre-2000 and post-2000. Fourthly, the heterogeneity, publication bias and threshold effect were not detected in the study by Squizzato et al. The finally noteworthy strength of our study was that the Fagan plot analysis was used for exploring the clinical utility of the TLS. Our results indicated when pretest probability was 1.3% which was defined by Wells’ Criteria, that a positive test result would increase the probability of PE to 8% and a negative test result would reduce the probability to 0%; When pretest probability was 16.2% a positive test result would increase the probability of PE to 55% and a negative test result would reduce the probability to 5%; When pretest probability was 40.6% a positive test result would increase the probability of PE to 81% and a negative test result would reduce the probability to 15%. Meanwhile, we found that when the pre-test probability was above 50% or below 10%, a positive or negative test result would accurately diagnose or exclude PE. In addition, a positive or negative TLS result could increase or reduce the probability of PE by nearly 30% among moderate risk populations. Therefore, the combination of TLS and other useful diagnostic tools such as the D-dimer test, echocardiography, lower extremity compression ultrasonography, EBUS and more objective and practical score tools may be the focuses of research in the future to reduce unnecessary CTPA [50], especially EBUS, which offers a complementary function with TLS, and performances very important roles in diagnosing central PE.

There are several limitations that deserve to be mentioned. Firstly, a significant heterogeneity was present which limited the robustness of the conclusions that were reached. Secondly, there was significant threshold effect in our study due to the various cut-off values across the studies and were not validated. Thirdly, TLS is an operator-dependent investigation, but not all studies in this meta-analysis made it clear that the level of their operators and this may be the potential source of significant heterogeneity in our study. Fourthly, the included patients in our meta-analysis may not be representative of the general population because of the high prevalence of PE in our study [51, 52]. Therefore, it is difficult to rule out the probability of potential selection bias.

## Conclusion

The present meta-analysis suggested that transthoracic lung ultrasonography is helpful in diagnosing pulmonary embolism. Although, the positive transthoracic lung ultrasonography results significantly increase the post-test probability of pulmonary embolism and the negative transthoracic lung ultrasonography results significantly decrease the post-test probability of pulmonary embolism, which may change some patients’ diagnostic processes, it is inappropriate to generally use transthoracic ultrasonography in diagnosing pulmonary embolism due to the lack of appropriate tools used to calculate the pre-test probability of pulmonary embolism.

## Supporting Information

S1 Checklist PRISMA Checklist for this meta-analysis. (DOC)

S1 Table Characteristics of Eligible Studies-2. (DOCX)
