# Supplementary material for: Role of Transthoracic Lung Ultrasonography in the Diagnosis of Pulmonary Embolism: A Systematic Review and Meta-Analysis
Source: PLoS One. 2015 Jun 15;10(6):e0129909. doi: 10.1371/journal.pone.0129909 (PMC4468196; doi:10.1371/journal.pone.0129909)
Supplement: S1 Table — (DOCX) [file pone.0129909.s002.docx]

| **TABLE 1.** **Characteristics of Eligible Studies-2** | | | |
| --- | --- | --- | --- |
| **Author** | **Inclusion criteria or exclusion criteria** | **Index test (****pre-defined ultrasound criteria)** | **Reference test** |
| Peiman（10） | Inclusion:  Age>18years; Suspected of having a PE;  Wells score >4 or D-dimer value ≥500ng/ml. | Pleural based well-demarcated echo-poor triangular or rounded consolidations of at least 0.5 cm in size. | MCTPA |
| Comert（24） | Inclusion:  High clinical suspicion: presence of risk factors and unexplained dyspnea, hypoxemia.  Moderate clinical suspicion: presence of risk factors and dyspnea/hypoxemia which can be explained by conditions other than PE or the presence of unesplained dyspnea/hypoxemia without risk factors. | 1. Two or more wedge-shaped, triangular, or rounded pleura-based hypoechoic lesions with/ without pleural effusion.  2. One wedge-shaped, triangular, or rounded pleura -based hypoechoic lesions with pleural effusion.  3. One wedge-shaped, triangular, or rounded pleura -based hypoechoic lesions .  4. Nonspecific subpleural lesions.  5. Normal sonographic findings.  1+2+3 PE was suggested.  4+5 PE was not supposed. | MCTPA |
| Pfeil（28） | Inclusion:  Patients presented typical symptoms of PE with acute onset of complaints . | NR | MCTPA |
| Mathis（23） | Inclusion:  Clinical suspicion of PE under consideration of the risk factors.  Exclusion:  Patients with deep vein thrombosis without PE symptoms. | 1. PE was considered when two or more characteristic triangular or rounded pleura-based lesions were demonstrated.  2. PE probable: one typical lesion with a corresponding low-grade pleural effusion.  3. PE possible: nonspecific subpleural lesions <5 mm in size or a single pleural effusion alone.  4. PE not established: normal chest sonography. | Single-slice CTPA or  at least two of the following criteria positive:highclinical suspicion; D-dimer ;  Proven leg vein thrombosis; Echocardiography;  V/Q scan;  Biopsy/necropsy. |
| Reissing（31） | Inclusion:  Patients presented with a typical history of PE with  acute onset of complaints including dyspnea, pleuritic chest pain, hemoptysis, vertigo or syncope, and/or tachypnoe. | NR | A diagnosis of PE was accepted, on direct computer tomographic visualisation of PE. If no direct visible emboli was seen on CT-scans,  diagnosis of PE was accepted, if (1) typical history and (2) a positive ventilation/perfusion  scanning as well as (3) an elevated D-dimer-level were  present. |
| Mohn（25） | Inclusion:  Recent clinical symptoms of PE had to be present.  Exclusion:  Patients with symptoms that had been reported more than 7 days previously. Patients who had a clinical indication of acute massive PE in the emergency department were referred to the Intensive Care Unit or unavailability of a 3-month followup. | PE suggestive:  1. Wedge-shaped, hypoechoic, homogeneous pleural -based lesions.  2. Sharply outlined pleural-based lesions, triangular or rounded to the hilus.  PE nonsuggestive:  1. Unspecific lesions of other shapes that have not been described in connection with PE.  2. No lesions detected.  3. The presence of an isolated pleural effusion. | 1. Clinical probability and high-probability lung scan.  2. Deep vein thrombosis shown by sonography.  3. Positive helical computed tomographic findings.  4. Pulmonary angiography. |
| Lechleitner（26） | Inclusion:  Clinical suspicion of PE.  Exclusion:  Haemodynamically unstable patients, those receiving mechanical ventilation, patients with contraindication to MRI, pregnant women or those wo declined to participate in the study. | Specific lesions:hypoechoic and homogenous, rounded and less sharply bordered to the ventilated lung or wedge-shaped, well demarcated lesions with a hyperechoic reflection at the centre.  Unspecific lesions:all lesions of other shapes, which have never been described in connection with PE.  No lesion: normal pleural reflex. | MRI-Angiography |
| Reissing（27） | Inclusion:  Patients had a typical history of PE with the acute onset of complaints:dyspnea, pleuritic chest pain, hemoptysis,vertigo or syncope, and/or tachypnea. | NR | Spiral CT scanning or at least three of the following positive results: typical history; echocardiography; venous duplex sonography or contrast venography of the legs; V/Q scanning;  D-dimer level. |
| Mathis（19） | Inclusion:  Patients with suspected pulmonary embolism. | NR | Spiral computed tomography (single-row), if sonograms  showed PE-typical subpleural lesions, diagnosis of PE was accepted if venous duplex sonography, echocardiography, and fibrin dimer tests or the pathologists supported the diagnosis. |
| Lechleitner（18） | Inclusion:  Patients with clinical signs of PE within 24 hours of onset of symptoms. | Identical with the above Lechleitner(2002). | V/Q lung scan  and D-dimer |
| Mathis （17） | Inclusion:  Patients with clinical signs of pulmonary embolism/infarction . | NR | Chest x-ray;  V/Q lung scan;  Pulmonary angiography;  Necropsy. |
| Kroschel（30） | Clinical suspicion of  PE. | Wedge-shaped echo-poor lesion  with or without a local effusion.  Possible PE: local effusion without  Ventilation defect | Perfusion lung scan (only high  probability and negative scan for PE). |
| Mathis（29） | Clinical signs of pulmonary infarction (pleuritic chest pain, dyspnea, cough and haemoptysis) | NR | Composite test:  Different combination of  chest X ray,  echocardiography, lung  scan, angiography, and/or  autopsy. |
| MCTPA, multi-detector computed tomography pulmonary angiography.  NR, not reported . | | | |
